# Grazing-induced microbiome alterations drive soil organic carbon turnover and productivity in meadow steppe

**DOI:** 10.1186/s40168-018-0544-y

**Published:** 2018-09-20

**Authors:** Weibing Xun, Ruirui Yan, Yi Ren, Dongyan Jin, Wu Xiong, Guishan Zhang, Zhongli Cui, Xiaoping Xin, Ruifu Zhang

**Affiliations:** 1 Jiangsu Provincial Key Lab for Organic Solid Waste Utilization, National Engineering Research Center for Organic-based Fertilizers, Jiangsu Collaborative Innovation Center for Solid Organic Waste Resource Utilization, Nanjing Agricultural University, Nanjing, 210095 China; 2 Key Laboratory of Microbial Resources Collection and Preservation, Ministry of Agriculture, Institute of Agricultural Resources and Regional Planning, Chinese Academy of Agricultural Sciences, Beijing, 100081 China; 3 National Hulunber Grassland Ecosystem Observation and Research Station, Institute of Agricultural Resources and Regional Planning, Chinese Academy of Agricultural Sciences, Beijing, 100081 China

**Keywords:** Temperate meadow steppe, Cattle grazing, Microbial composition, Soil incubation, SOC-decomposition enzymatic activity, Soil productivity

## Abstract

**Background:**

Grazing is a major modulator of biodiversity and productivity in grasslands. However, our understanding of grazing-induced changes in below-ground communities, processes, and soil productivity is limited. Here, using a long-term enclosed grazing meadow steppe, we investigated the impacts of grazing on the soil organic carbon (SOC) turnover, the microbial community composition, resistance and activity under seasonal changes, and the microbial contributions to soil productivity.

**Results:**

The results demonstrated that grazing had significant impacts on soil microbial communities and ecosystem functions in meadow steppe. The highest microbial α-diversity was observed under light grazing intensity, while the highest β-diversity was observed under moderate grazing intensity. Grazing shifted the microbial composition from fungi dominated to bacteria dominated and from slow growing to fast growing, thereby resulting in a shift from fungi-dominated food webs primarily utilizing recalcitrant SOC to bacteria-dominated food webs mainly utilizing labile SOC. Moreover, the higher fungal recalcitrant-SOC-decomposing activities and bacterial labile-SOC-decomposing activities were observed in fungi- and bacteria-dominated communities, respectively. Notably, the robustness of bacterial community and the stability of bacterial activity were associated with α-diversity, while this was not the case for the robustness of fungal community and its associated activities. Finally, we observed that microbial α-diversity rather than SOC turnover rate can predict soil productivity.

**Conclusions:**

Our findings indicate the strong influence of grazing on soil microbial community, SOC turnover, and soil productivity and the important positive role of soil microbial α-diversity in steering the functions of meadow steppe ecosystems.

**Electronic supplementary material:**

The online version of this article (10.1186/s40168-018-0544-y) contains supplementary material, which is available to authorized users.

## Background

Grazing by livestock is one of the most extensive land uses and occupies more than one third of the global land area [1]. Overgrazing has been reported to reduce floristic diversity and biomass and is probably the most pervasive and significant process of degradation in grasslands [2]. The importance of floristic diversity in driving the productivity and other ecosystem functions has been demonstrated by many studies [3, 4]. However, the importance of floristic diversity has been questioned recently [5, 6]. Some of these queries suggest that the productivity of many terrestrial ecosystems depends on the availability of resources [7–9]. In particular, soil microbes are important components in maintaining ecosystem functions and enhance soil productivity due to their critical roles, including litter decomposition, biogeochemical nutrient cycling, soil agglomeration, and fertility promotion [10–12]. Therefore, it is important to promote the transition from above-ground studies to below-ground ones to increase our understanding of the soil microbial behaviors. These studies may provide ecologists with insights into the seemingly divergent results observed above the ground. To date, although some investigations have studied top-down interactions [13–15], our knowledge of the mechanisms by which soil microbial communities and ecosystems function to maintain soil productivity is limited, especially the grazing-induced changes in below-ground communities [16].

It has been well established that both the composition and functional capabilities of soil microbial community can be strongly influenced by environmental variables such as climate [17], vegetation [18], and soil conditions [19]. In steppe ecosystems, grazing is a key regulator that can directly or indirectly affect the abovementioned environmental variables [20, 21] and then affect the diversity and composition of soil microbial community [22], resulting in the alteration of the functional performance and nutrient provision patterns in soil [23]. Indeed, some studies have demonstrated that grazing has a significant effect on soil microbial community and on soil carbon (C) and nitrogen (N) availability [24–26]. It has also been suggested that different grazing intensities may alter the distribution of soil bacteria and fungi and affect soil respiration [27, 28]. Despite these findings, few studies have been conducted on the effect of grazing on below-ground microbial community and on the C sequestration capacity and productivity of soil in grassland ecosystems [29, 30]. Therefore, these integrated top-down and bottom-up interactions need to be further investigated.

The Inner Mongolian grassland is an important component of the Eurasian grassland, and the grassland of Hulunber, which is at the east edge of Eurasian grassland, is the most representative temperate meadow steppe, with high soil fertility and biodiversity [31]; this grassland can undergo a loss of species under high grazing intensity and is favorable for our research. Therefore, to explore the impacts of grazing on soil microbial community and consequent on soil productivity, a long-term grazing-intensity-gradient experiment was established in 2009 at the Hulunber Grassland Ecosystem Research Station (HGERS) of the Chinese Academy of Agricultural Sciences (CAAS) in Hulunber, Inner Mongolia, China. 16S and internal transcribed spacer (ITS) regions of the rRNA gene amplicon sequencing were performed to evaluate the structure of soil microbial community. A variety of SOC-decomposing enzymatic activities were performed to evaluate the microbial activity on SOC turnover. The objectives of this study were to investigate grazing-induced changes in the composition, diversity, activity, and stability of soil microbial community and to study the microbial contributions to SOC turnover and productivity.

## Methods

### Study design

The experimental site was located at the Hulunber Grassland Ecosystem Research Station (HGERS) of the Chinese Academy of Agricultural Sciences (CAAS) (119°94′~119°96′ E, 49°32′~49°34′ N) in Hulunber, Inner Mongolia, China. The soil at the station is kastanozems according to FAO/Unesco System of Soil Classification. This region covers a semi-arid continental climate with a mean annual temperature range of almost − 3 °C and annual precipitation range of 350~400 mm. The vegetation in this region is characterized as typical meadow steppe. *Leymus chinensis*, *Stipa baicalensis*, *Carex duriuscula*, *Galium verum*, *Bupleurum scorzonerifolium*, and *Filifolium sibiricum* are the dominant plant species. This grassland was subjected to six levels of cattle grazing intensity in a randomized complete block design (Additional file 1: Figure S1). Four of these six grazing intensities with 0.00, 0.42, 0.83, and 1.67 cattle ha^−1^, corresponding to 0, 2, 4, and 8 cattle per plot, were selected for this study, and these grazing intensities were designated G0, G2, G4, and G8, respectively. All these sites have been used as summer pastures, with a grazing period from June to September, since 2009. Rest grazing begins in October and ends in May of the following year.

### Soil sample collection and analysis

Soil samples were collected in June (designated J0, J2, J4, and J8 for soil samples collected from treatments G0, G2, G4, and G8, respectively) and August (designated A0, A2, A4, and A8 for soil samples collected from treatments G0, G2, G4, and G8, respectively) of 2015. To allow the detection of both α- and β-diversity, a nested sampling approach was established [32] in which all sampling sites were located on concentric circles with radii of 150 m, 15 m, 1.5 m, and 0.15 m in a plot. With this sampling strategy, a total of 17 samples were collected from each plot (Additional file 2: Figure S2). Soil samples were obtained from the upper 20 cm (the litter layer was removed) of the plots in soil cores with diameters of 10 cm. All samples were sieved, homogenized, and subdivided using standard methods. Soils for measuring physiochemical properties were air-dried, soils for molecular analyses were stored at − 80 °C until extraction of deoxyribonucleic acid (DNA), and soils for establishing microcosms were stored at room temperature. The edaphic properties of all soil samples were evaluated at the Qiyang Red Soil Experimental Station of the CAAS, and the detailed methods used were described previously [33].

### Microcosm incubation

In this field experiment, different grazing intensities result in different plant coverage, which leads to differences in the soil water content of the plots (Additional file 3: Table S1) and influences the soil temperature. The water content and temperature of soil are important factors affecting C decomposition in soil. Thus, given the grazing-induced differences in water content and temperature conditions in situ and to provide an accurate assessment of the effects of grazing on the potential SOC decomposition rate, we established soil microcosms and incubated these microcosms under different water content and temperature conditions. The water content of G0 (22%) was defined as 100% of the field water content; 75% and 50% field water contents were chosen for moisture perturbance. A temperature gradient (24 °C, 33 °C, and 42 °C, which is consistent with changes in temperature between June and August) was used for temperature perturbance.

Soil microcosms were prepared as described by Xun et al. [33]. Eighteen replicate microcosms were established for each soil sample (thus, we have 18 microcosm replicates × 17 soil samples × 3 plot replicates = 918 microcosms for each grazing intensity), and each microcosm was constructed by placing 150 g of fresh soil into a 250-mL bottle. Sterile water was added to maintain the moisture level at 100% of the field water content, and the microcosms were preincubated at 24 °C in the dark. After preincubation for 1 week, 16 of these 18 replicate microcosms were randomly chosen for moisture and/or temperature perturbance testing, while the remaining 2 microcosms were incubated as before (control, no perturbance, moisture at 100% of the field water content, and temperature of 24 °C). The detailed incubation conditions are described in Additional file 4: Table S2. The incubation process lasted for 2 months.

### Enzymatic activity analyses

The following enzyme activity assays were conducted: (i) the activities of invertase (EC 3.2.1.26), maltase (EC 3.2.1.20), amylase (EC 3.2.1.1), xylanase (EC 3.2.1.8), cellulose (EC 3.2.1.4), and pectinesterase (EC 3.1.1.11) were measured using the 3,5-dinitro-salicylic acid colorimetric method with sucrose, methyl polyglycoside, starch, xylan, carboxymethylcellulose and pectin as substrates, respectively; (ii) the activity of β-glucosidase (EC 3.2.1.21) was measured using an improved colorimetric method [34] with *p*-nitrophenyl glycoside as the substrate.

### DNA extraction, qPCR, and amplicon sequencing

Total DNA was extracted from 0.25 g of soil using a PowerSoil DNA Isolation Kit (Mo Bio Laboratories Inc., Carlsbad, CA, USA). To minimize the DNA extraction bias, three successive DNA extractions of each soil sample were pooled before performing polymerase chain reaction (PCR). A NanoDrop ND-2000 spectrophotometer (NanoDrop, ND2000, Thermo Scientific, Wilmington, USA) was used to assess DNA quality on the basis of the 260/280 nm and 260/230 nm absorbance ratios. Extracted DNA was stored at − 20 °C until use.

Bacterial and fungal abundances were determined by quantitative PCR (qPCR) analysis using the Power SYBR Green PCR Master MIX (Biosystems, Warrington, UK) on an ABI 7500 Real-Time PCR System (Applied Biosystems, CA, USA). The following primer sets were used: F347 (5′-GGAGGCAGCAGTRRGGAAT-3′) and R531 (5′-CTNYGTMTTACCGCGGCTGC-3′) (194 bp) for bacterial 16S rRNA gene abundance and FungF (5′-GTAGTCATATGCTTGTCTC-3′) and FungR (5′-ATTCCCCGTTACCCGTTG-3′) (346 bp) for fungal 18S rRNA gene abundance.

The V4 hypervariable region of the bacterial 16S rRNA gene was amplified using the primers 515F (5′-GTGCCAGCMGCCGCGGTAA-3′) and 806R (5′-GGACTACHVGGGTWTCTAAT-3′) to assess bacterial communities. The internal transcribed spacer (ITS2) region of the rRNA gene of fungi was amplified using the primers ITS3 (5′-GCATCGATGAAGAACGCAGC-3′) and ITS4 (5′-TCCTCCGCTTATTGATATGC-3′) to assess fungal communities. The PCR amplicons were combined in equimolar ratios, and sequencing was conducted by Bion Biotechnology Co., Ltd. (Nanjing, China) on an Illumina MiSeq platform with separate sequencing runs for the 16S and ITS rRNA gene amplicon pools. The sequencing data were processed using the UPARSE pipeline (http://drive5.com/usearch/manual/uparse_pipeline.html) [35]. The raw sequences were subjected to quality control. The singleton and chimeric sequences were removed after dereplication, and the remaining sequences were categorized into operational taxonomic units (OTU) with 97% similarity and then assigned taxonomy using the Silva database (Release 128) (https://www.arb-silva.de/) and the UNITE database (Release date: August 2016) (https://unite.ut.ee/) for the 16S and ITS rRNA genes, respectively. The 16S and ITS rRNA gene sequences are available at the NCBI Sequence Read Archive under the accession numbers SRP117970 and SRP119882.

### Data analyses

The sequencing data were analyzed as follows: (i) the percentage of each taxonomy was designated the relative abundance, (ii) taxonomic α-diversity was calculated as the OTU richness and Shannon diversity of a single sample site, (iii) taxonomic γ-diversity was calculated as the OTU richness of an experimental plot, and (iv) β-diversity (phylogenetic community dissimilarity) was calculated using FastUnifrac [36]. Duncan’s multiple comparison test was used to calculate the statistical significance among samples. Tukey’s HSD test was used to calculate the statistical significance between two samples. Correlations were calculated using Mantel tests and Spearman correlations. All statistical analyses were performed with the Vegan package (v.2.4-1) [37] in R software (version 3.3.2).

## Results

### Intensification of grazing triggers fast-growing and bacteria-dominated communities

**Fig. 1:**
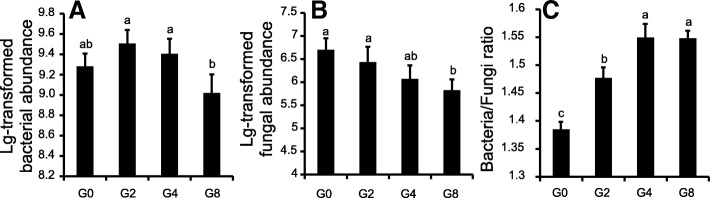
**a** Lg-transformed bacterial abundance, **b** Lg-transformed fungal abundance, and **c** bacteria/fungi ratio at all grazing intensities. Statistical analyses were performed independently for bacterial abundance, fungal abundance, and bacteria/fungi ratio using Duncan’s multiple comparison test. The results were shown with letters a to c

Grazing had a significant impact on soil bacterial and fungal abundance, whereas the seasonal changes did not. The bacterial abundance was consistently higher in G2 and G4 and lower in G8 (Fig. 1a). However, the fungal abundance was highest in G0 and decreased with increasing grazing intensity (Fig. 1b). Overgrazing (G8) led to significant reductions in both bacterial and fungal abundance. When the bacteria/fungi ratios were calculated, we observed that higher bacteria/fungi ratios were associated with higher grazing intensities, indicating that bacteria become prevalent with increasing grazing intensity (Fig. 1c).

Our sequencing results indicated significant differences in the bacterial and fungal communities responding to cattle grazing. The strongest distinctions were observed between the below-moderate (G0 and G2) and moderate-to-high (G4 and G8) grazing intensities. With regard to soil bacteria (Additional file 5: Figure S3A), most slow-growing bacteria of the phyla *Acidobacteria*, *Chloroflexi*, *Planctomycetes*, and *Verrucomicrobia* were more abundant in G0 and G2. However, most fast-growing bacteria of the phyla *Bacteroidetes*, *Firmicutes*, *Nitrospira*, and *Proteobacteria* were more abundant in G4 and G8. For soil fungi (Additional file 5: Figure S3B), the most abundant fungal phyla were *Ascomycota*, *Basidiomycota*, *Glomeromycota*, and *Zygomycota*. Among them, *Ascomycota* was the most prevalent phylum, with a relative abundance ranging from 80.6 to 89.9%. Moreover, the identified classes from the phyla *Glomeromycota* and *Zygomycota* and several classes belonging to *Ascomycota* (*Geoglossomycetes*, *Pezizomycetes*, *Sordariomycetes*, etc.) were more abundant in G0 and G2. Notably, seasonal changes had less impact on microbial composition than grazing.

### Grazing affects the diversity and robustness of microbial communities

**Fig. 2:**
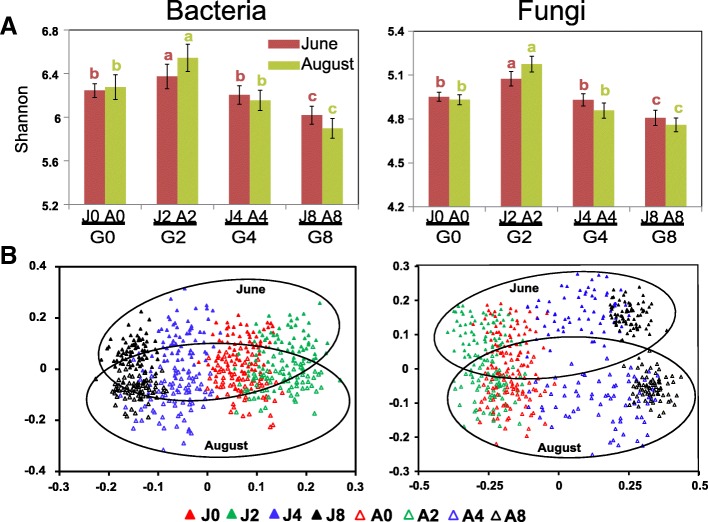
**a** Bacterial and fungal α-diversity (Shannon index) in June and August. Statistical analyses were performed independently for Shannon diversity at two seasons using Duncan’s multiple comparison test. The results were shown with colored letters a to c. **b** Non-metric multidimensional scaling (NMDS) analysis for bacterial and fungal communities

Soil microbial diversity was estimated using α-diversity (species richness at sample site level), γ-diversity (species richness at plot level), and β-diversity (the community differentiation among sample sites in one plot) simultaneously. First, light grazing intensity (G2) resulted in the highest soil microbial α-diversity, while high grazing intensity (G8) resulted in the lowest α-diversity, the significance of which is indicated by the Shannon index (Fig. 2a) and OTU richness (Additional file 6: Figure S4A) of the samples. Meanwhile, the sites with moderate grazing intensity (G4) possessed the same level of microbial α-diversity as G0. Second, microbial γ-diversity (Additional file 6: Figure S4B) was observed to be the highest in G2 and the lowest in G8. However, G4 had the same level of species richness as G2 at plot level. Third, the β-diversity of the microbial communities were calculated using the unweighted (Additional file 6: Figure S4C) and weighted (Additional file 6: Figure S4D) Unifrac distances, and the communities of G4 showed the greatest β-diversity, followed by the communities of G2, G0, and G8.

A non-metric multidimensional scaling (NMDS) analysis was performed to compare the microbial communities. Bacterial communities were separated across the first principal coordinate on the basis of grazing intensity (Fig. 2b). The pattern of separation is consistent with a gradient of grazing intensity from G0 and G2, across moderate grazing intensity (G4), and to high grazing intensity (G8). In addition, the communities were separated by seasonal variation across the second principal coordinate, indicating that soil bacterial communities also respond to seasonal changes. Moreover, a similar separation pattern was observed for fungal communities.

**Fig. 3:**
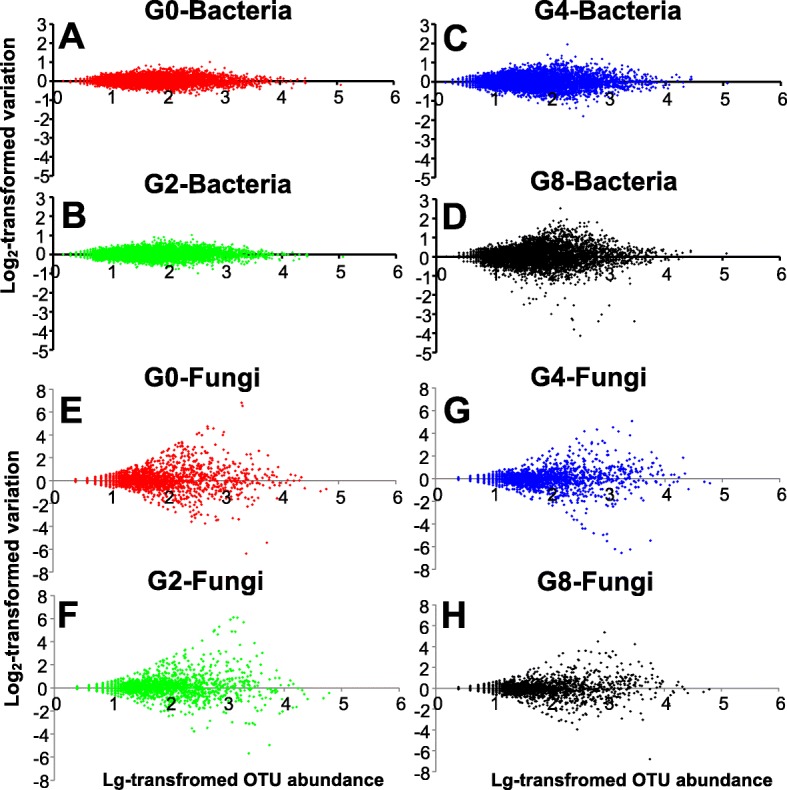
Seasonal variations of normalized OTU abundances between June and August for every grazing intensity. **a** Seasonal variations of bacterial community in G0. **b** Seasonal variations of bacterial community in G2. **c** Seasonal variations of bacterial community in G4. **d** Seasonal variations of bacterial community in G8. **e** Seasonal variations of fungal community in G0. **f** Seasonal variations of fungal community in G2. **g** Seasonal variations of fungal community in G4. **h** Seasonal variations of fungal community in G8

Grazing has significant effects on grassland microbial community composition and diversity, which may alter the robustness of microbial community. Indeed, community robustness is related to its responses to seasonal changes. Therefore, we conducted an OTU differential analysis using the normalized OTU relative abundances and performed a logarithmic ratio test between the soil samples collected at each level of grazing intensity in June and August to determine the community robustness. As indicated by the broom-shaped “tails,” where more divergent “tails” indicate weak robustness, the seasonal variations of the bacterial communities of G8 (Fig. 3d) were greater than those of the communities of G4 (Fig. 3c), followed by G2 (Fig. 3b) and G0 (Fig. 3a), which indicated that the stability of bacterial community decreased with increasing grazing intensity. In comparison, the seasonal variations in the fungal communities fluctuated considerably across all grazing intensities (Fig. 3e to 3h).

### Grazing alters SOC decomposition and functional stability

To study how soil SOC turnover responds to seasonal changes, a soil incubation experiment was established by varying the water content and temperature conditions (moisture and temperature were determined by field investigation). Various soil enzymatic activities, including those of SOC-decomposing enzymes ranging from those involved in labile- to recalcitrant-SOC decomposition, were detected.

We found that the soil enzymatic activities were different across incubation conditions, whereas the microbial abundances were not (qPCR data not shown). Intriguingly, the activities of the labile-SOC-decomposing enzymes (invertase, maltase, and amylase) exhibited positive relationships (*R* ≥ 0.597, *P* ≤ 0.019) with bacterial abundance but weak relationships (*R* ≤ 0.286, no significance) with fungal abundance (Table 1). However, the activities of the recalcitrant-SOC-decomposing enzymes (pectinesterase, cellulase, and xylanase) exhibited positive relationships (*R* ≥ 0.659, *P* ≤ 0.009) with fungal abundance but weak relationships (*R* ≤ 0.262, no significance, except *P* = 0.035 for xylanase) with bacterial abundance. Additionally, the activity of the enzyme β-glucosidase was positively correlated with both bacterial (*R* = 0.628, *P* = 0.021) and fungal (*R* = 0.585, *P* = 0.015) abundances.

**Table 1:** Spearman’s rank correlations of SOC-decomposing enzyme activities with bacterial and fungal abundances

	Labile-SOC decomposition → recalcitrant-SOC decomposition						
	Invertase	Maltase	Amylase	β-glucosidase	Xylanase	Cellulase	Pectinesterase
Bacterial abundance (lg)	0.600 (< 0.001)	0.657 (< 0.001)	0.597 (0.019)	0.628 (0.021)	0.257 (0.035)	0.227 (n.s.)	0.262 (n.s.)
Fungal abundance (lg)	0.038 (n.s.)	0.167 (n.s.)	0.286 (n.s.)	0.585 (0.015)	0.742 (< 0.001)	0.659 (0.009)	0.757 (< 0.001)

*lg* log_10_ transformation, *n.s.* not significant

To provide a reasonable assessment of microbial activity, the ratios of enzymatic activity to microbial abundance were calculated (only the significantly correlated activities and microbial abundances were used for the calculations). Soil microbial activity responded differently to moisture and temperature perturbances; bacterial activity was detected to be the highest in G4 and lowest in G8 under temperature or water content gradient (Additional files 7 and 8: Figures S5 and S6). However, fungal activity was highest in G0 and lowest in G8 under temperature or water content gradient (Additional files 9 and 10: Figures S7 and S8). Meanwhile, the fungal activity seemed to be more sensitive than bacterial activity because of the high variability of fungal activity under moisture and temperature perturbances at the same grazing intensity.

**Fig. 4:**
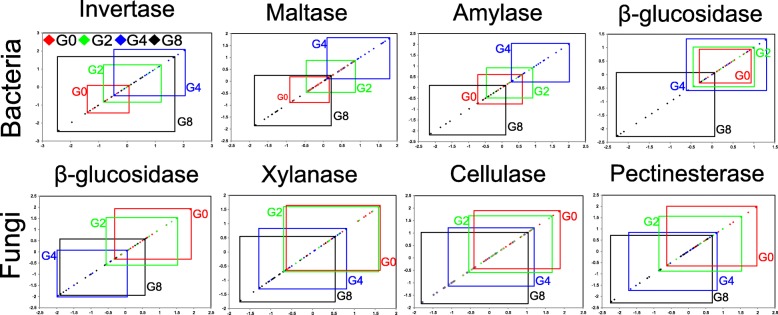
Normally distributed microbial activity under various incubation conditions. The position and size of the rectangle indicate activity and stability. The right-most rectangle indicates higher activity. The smaller-sized rectangle indicates more stable activity. Only the significantly correlated activities and microbial abundances were considered for calculation

A normal distribution of microbial activity was performed to demonstrate the stability of microbial activity under various water and temperature conditions (Fig. 4). The right-most rectangle indicated higher activity, and the smaller-sized rectangle indicated more stable microbial activity under perturbances. In this analysis, the highest bacterial activity was observed in G4, whereas the most stable bacterial activity was found in G0. Moreover, the strongest individual predictor of bacterial activity was bacterial β-diversity (*R* = 0.794, *P* = 0.002), and soil bacterial activity was also significantly related to soil pH (*R* = 0.726, *P* = 0.004), bacterial γ-diversity (*R* = 0.692, *P* = 0.034), and bacterial α-diversity (*R* = 0.625, *P* = 0.045) (Table 2). However, the highest fungal activity was observed in G0, and the large variations in fungal activity indicated that fungal activity is sensitive to seasonal changes rather than to cattle grazing. In addition, the strongest individual predictor of fungal activity was fungal abundance (*R* = 0.829, *P* = 0.001), and soil fungal activity was also significantly related to fungal α-diversity (*R* = 0.571, *P* = 0.039) and SOC concentration (*R* = 0.507, *P* = 0.037).

**Table 2:** Spearman’s rank correlations of microbial activity with microbial diversity, abundance, soil pH, and SOC concentration (estimated using Mantel tests)

	Bacterial activity^a^ (Labile-SOC-degrading efficiency)		Fungal activity^b^ (Recalcitrant-SOC-degrading efficiency)	
	Mantel statistic *R*	Significance (*P*)	Mantel statistic *R*	Significance (*P*)
Bacterial α-diversity	0.625	0.045	–	–
Bacterial γ-diversity	0.692	0.034		
Bacterial β-diversity	0.794	0.002	–	–
qPCR (bacteria)	0.518	0.053	–	–
Fungal α-diversity	–	–	0.571	0.039
Fungal γ-diversity			0.483	0.044
Fungal β-diversity	–	–	0.267	0.227
qPCR (fungi)	–	–	0.829	0.001
pH	0.726	0.004	0.304	0.265
SOC	0.218	0.240	0.507	0.037

^a^Bacterial activity, estimated using the activity of invertase, maltase, and amylase

^b^Fungal activity, estimated using the activity of xylanase, cellulase, and pectinesterase

### Soil productivity is related to microbial α-diversity

**Fig. 5:**
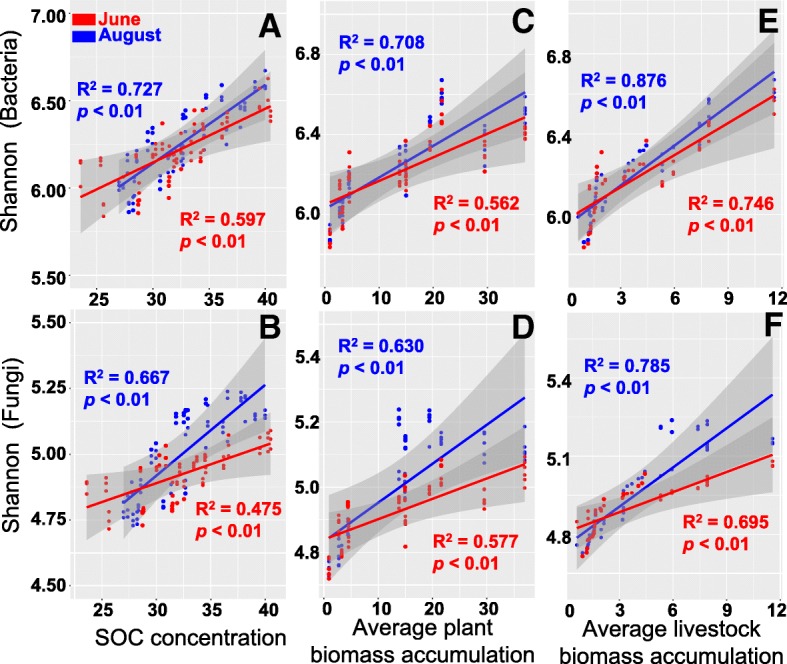
Linear regression analysis between microbial α-diversity (Shannon index) and SOC concentration (**a** bacteria; **b** fungi), average plant biomass accumulation (**c** bacteria; **d** fungi), and average livestock biomass accumulation (**e** bacteria; **f** fungi)

Soil productivity was estimated using both below-ground SOC concentrations and above-ground plant and livestock biomass. We observed weak correlations between microbial activity and soil productivity based on the results of Mantel tests. However, linear regression analysis results revealed that bacterial (*R*^2^ ≥ 0.597, *P* < 0.01) and fungal (*R*^2^ ≥ 0.476, *P* < 0.01) Shannon diversity indices had significant positive correlations with SOC concentrations in both June and August (Fig. 5a, b). Meanwhile, we found notable significant positive correlations between microbial Shannon diversity and average plant biomass accumulation (*R*^2^ ≥ 0.562, *P* < 0.01 for bacteria, Fig. 5c; and *R*^2^ ≥ 0.577, *P* < 0.01 for fungi, Fig. 5d) and between microbial Shannon diversity and average livestock biomass accumulation (*R*^2^ ≥ 0.746, *P* < 0.01 for bacteria, Fig. 5e; and *R*^2^ ≥ 0.695, *P* < 0.01 for fungi, Fig. 5f).

## Discussion

### Grazing shifts microbial communities from fungi dominated and slow growing to bacteria dominated and fast growing

Different grazing intensities have direct and indirect impacts on soil environmental conditions and microbial community [38]. Livestock feed on aboveground plants and return nearly half of it as feces [39], which favors bacterial growth. Nevertheless, at overgrazing site, much trampling leads to high volume weights of soil [40], and lack of plant coverage induces drastic fluctuations in soil moisture and temperature, which results in a difficult environment for the growth of soil microorganisms. Therefore, we observed a leaping change in bacterial abundance. In addition, the increasing bacteria/fungi ratio suggested that bacteria became the dominant microorganisms while soil fungal abundance decreased at higher grazing intensities, which is probably because fungi favor less perturbed ecosystems [41].

With regard to the effects of grazing on the composition of microbial community, we observed that different microbial taxa exhibited different behaviors. Many Basidiomycotal groups are sensitive to environmental perturbances and slow-growing due to the long-lasting dikaryotic state [42], and could be used as a fungal indicator of perturbance intensity. Moreover, the phylum *Glomeromycota*, members of which are known as arbuscular mycorrhizal fungi (AMF), is closely associated with plant biomass [43]. Therefore, conditions of intensive grazing are not conducive to colonization by these fungi [44] due to enhanced soil perturbance and decreased plant richness and biomass.

Particularly, although the aboveground plants are nearly half-returned as feces, limited herbage under intensive grazing may impel the cattle to consume more energy foraging around the site, thus will increase the aboveground herbage consumption and belowground nutrient turnover, and consequently decrease the SOC concentration [31]. However, in this site, intensive grazing increased the relative abundances of *Bacteroidetes*, *Proteobacteria*, *Nitrospria*, etc. These abundant bacterial taxa are mostly copiotrophic bacteria [45], which are generally fast growing and positively correlated with SOC concentrations [46]. This result was initially surprising, as a previous study revealed that intensive grazing induces the proliferation of gram-positive bacteria [47], which was confirmed only on the bacterial phylum *Firmicutes* at the highest grazing intensity in this study. We considered that the feces contain more available nutrient and labile organic substrates than plant residues, hence promote the growth of copiotrophic bacteria. Taken together, these findings suggest that grazing shifts the microbial communities from slow growing and fungi dominated to fast growing and bacteria dominated.

### Grazing alters soil bacterial and fungal diversity simultaneously but has different impacts on the robustness of bacterial and fungal community

Grazing probably modulates the diversity of soil microbes by altering the competitive interactions between dominant microbes and releasing/suppressing subordinate microbes [16]; however, overgrazing may have negative effects on microbial diversity. In this study, the microbial α-diversity of G0 and G4 were both significantly lower than that of G2, but the γ-diversity of G4 was at the same level as that of G2. These data indicated that light grazing had positive effects on soil microbial species richness at local and regional scale. However, moderate grazing decreased microbial species richness at local scale but had little impact at regional scale. These variations in species richness at different scales in moderate grazing intensity lead to a high level of β-diversity [48]. Indeed, we confirmed the observation using both unweighted and weighted Unifrac distances. Moreover, this observation was consistent with previous findings by Cline et al. [49], who carried out a long-term ungulate foraging intensity experiment and indicated that foraging intensity is associated with decreased bacterial richness (α-diversity) and increased distinct bacterial communities (β-diversity). They also predicted that high foraging intensity would lead to larger reductions in soil biological α- and β-diversity. Particularly, Griffiths et al. [50] suggested that these kinds of variations in diversity mainly correspond to greater environmental variation. We propose that these findings may be attributed to selective feeding, which causes alterations of nutrient supply and demand pattern at local scale, leading to greater environmental heterogeneity at regional scale.

In addition to the strong influence of grazing, seasonal changes affect soil microbial community synchronously [17]. As indicated by the broom-shaped “tails” in Fig. 3, increased variations of soil bacterial community were observed with increasing grazing intensity. Since soil microbes are susceptible to soil moisture and temperature [51] and many other characteristics [52], these differences were probably attributable to seasonal changes. For instance, grazing leads to a decrease in plant coverage [53], which induces rapid loss of soil moisture [54] and sharp fluctuations of soil temperature [55]. Therefore, intensive grazing makes the soil more susceptible to seasonal fluctuations and decreases soil microbial diversity, and thus, intensive grazing may decrease community robustness. However, in contrast to the bacterial communities, large variations were observed for fungal communities across all grazing intensities. We propose that plant growth may be the major factor responsible for the seasonal alterations in fungal community. This is consistent with Millard and Singh [56] that demonstrated that bacterial communities are more influenced by soil characteristics, while fungal communities are primarily influenced by vegetation.

### Microbial composition and robustness determine the SOC decomposition activity and stability

Soil bacteria and fungi have different impacts on SOC pool; hence, grazing influences the size and composition of SOC fractions via altering the microbial abundance and composition in soil [57]. Generally, intensive grazing lands require more available nutrients for plant growth. Therefore, soil microbes are forced to form a community, which exhibits higher nutrient-turnover rates. To date, we know that intensive grazing leads to bacteria-dominated food webs in soil [58, 59]. Our results are consistent with these observations, showing that bacteria, especially fast-growing copiotrophic bacteria, become dominant in intensive grazing lands. We detected the activities of several SOC-decomposing enzymes to provide an overview of SOC decomposition capacity. The distinctive relationships between SOC-decomposing enzyme activity and microbial abundance revealed that fungi are more important in recalcitrant-SOC turnover while bacteria are more important in labile-SOC turnover. For instance, fungi are more efficient at decomposing lignocellulose than bacteria [60]. Moreover, more copiotrophic bacteria contain more labile-SOC-decomposing genes [61], which promotes labile-SOC turnover. Therefore, the dominance of soil bacteria or fungi regulates SOC decomposition.

To provide a proper characterization of average microbial activity, we calculated the ratios of enzymatic activity to microbial abundance. This ratio is similar to the metabolic quotient (qCO_2_), which is defined as the respiration rate per unit of biomass [62]. We observed that soil bacterial activity (labile-SOC decomposition) was highest in G4, suggesting that moderate grazing enhanced whereas overgrazing suppressed the bacterial activity and ability to decompose labile SOC. On the other hand, soil fungal activity (recalcitrant-SOC decomposition) was highest in the non-grazed site (G0), suggesting that the introduction of grazing suppressed soil fungal activity and the ability to decompose recalcitrant SOC. This finding supported the hypothesis that bacteria and fungi are relatively more important decomposers in intensively grazed and lightly grazed grasslands, respectively.

We observed that the best parameter for predicting bacterial activity was bacterial β-diversity, followed by soil pH. It is apparent that soil pH has a strong effect on bacterial survival and activity [33]; however, there are few reports on the effects of bacterial β-diversity. We believe that the high bacterial activity in G4 was mainly due to the high proportion of copiotrophic bacteria. Therefore, moderate grazing will promote the growth of copiotrophic bacteria and enhance bacterial activity in meadow steppe. However, the activity of copiotrophic bacteria will be suppressed in overgrazed lands. Unlike bacterial community, weak relationship was found between fungal community and soil pH [19], and subsequent studies proved that soil fungal diversity and composition are related to SOC [63, 64], which is related to plant growth in grasslands. Moreover, Keiblinger et al. [65] suggested that the SOC-decomposition efficiency of fungi is more sensitive to vegetation and requires a high fungal biomass. Therefore, the association between fungal abundance and recalcitrant-SOC decomposition efficiency may act as a good predictor for fungal activity in natural habitats.

We also detected great fluctuations in bacterial activity at intensive grazing sites and in fungal activity across all grazing sites, which were related to the robustness of bacterial and fungal communities, respectively. Our findings were consistent with a previous result, which showed that microbial robustness determines the stability of soil functions [66]. Importantly, although we did not find significant changes in fungal abundance under various incubation conditions, fungal activity showed the same fluctuation tendencies, indicating that fungal activity is also sensitive to moisture and temperature. Taken together, these findings indicate that soil bacterial activity mainly lays in the abundant copiotrophic taxa and soil properties, and these abundant copiotrophic taxa are primarily responsible for the efficiency of labile-SOC decomposition. In addition, fungal activity is mainly affected by fungal abundance and plant growth and is primarily responsible for the efficiency of recalcitrant-SOC decomposition.

### Soil microbial α-diversity rather than SOC turnover rate predicts soil productivity

Generally, floristic complexity determines soil productivity in the absence of the interference of natural ecological systems [3, 67]. Ecologists have been trying to predict ecosystem services x(e.g., soil productivity) using floristic diversity in human- or herbivore-disturbed ecosystems, but the results have been mixed [3, 5, 7]. Van Der Heijden et al. [68] summarized that closed nutrient cycling occurs in less-perturbed soils with fungi-dominated food webs and that these soils usually support slow plant growth and low net primary productivity. Nevertheless, we found that moderate grazing-induced bacteria-dominated food webs and faster labile-SOC turnover rate did not achieve the highest productivity either. We conjectured that in bacteria-dominated food webs, rapid nutrient cycling occurs to provide suitable conditions for the growth of fast-growing bacteria, which may shift the SOC pool from C sinks to C sources and may suppress plant growth and productivity. Herein, strong positive effects of microbial α-diversity on soil C storage and productivity were detected, suggesting that there may be an internal microbiological mechanism to increase the proportions of photoassimilates in the soil C pool and food chain with higher microbial α-diversity rather than higher nutrient turnover rates. Thus, soil management intensity and associated changes in soil microbial community can steer the multiple functions of ecosystems and trade-offs between human demands, and ecosystem functions still need to be concerned [69].

## Conclusions

**Fig. 6:**
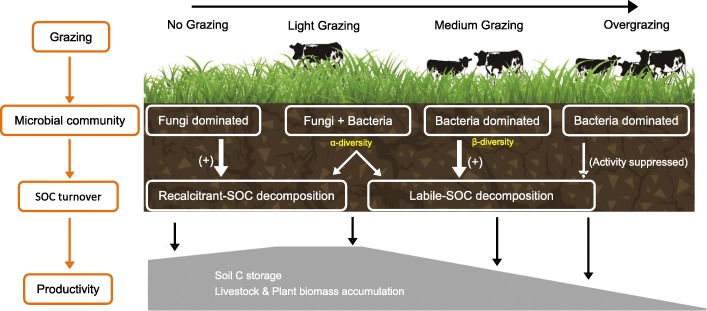
An overview of the influence of grazing on soil microbial community and soil productivity

The data presented here demonstrated that different levels of grazing had significant impacts on soil microbial communities and ecosystem functions in meadow steppes (Fig. 6). Grazing shifted microbial compositions from slow-growing and fungi-dominated to fast-growing and bacteria-dominated communities. We observed that fungal community was mainly responsible for recalcitrant-SOC decomposition and that fungal activity was positively correlated with fungal abundance. On the other hand, bacterial community was primarily responsible for labile-SOC decomposition, and bacterial activity was associated with β-diversity. However, microbial activity was suppressed in overgrazing soil. Particularly, soil productivity was not associated with bacterial or fungal activity but with microbial α-diversity. Thus, we argue that grazing affects soil productivity by regulating soil microbial community and nutrient turnover and that great soil productivity is principally determined by microbial α-diversity but does not require a very high microbial activity in meadow steppes.

## Additional files

Additional file 1: **Figure S1.** Sketch map of the experimental site. (PDF 133 kb)

Additional file 2: **Figure S2.** Sampling scheme. A total of 17 soil cores (red points in the diagram) were taken from each plot. (PDF 18 kb)

Additional file 3: **Table S1.** Soil water content (%) at sampling times (June and August, 2015). (DOCX 16 kb)

Additional file 4: **Table S2.** Detailed soil incubation conditions for moisture and/or temperature perturbance testing. (DOCX 16 kb)

Additional file 5: **Figure S3.** Heatmap for (A) bacterial and (B) fungal communities. Color scale from greatest (red) to lowest (green) relative abundances within rows. Only the classifiable microbial classes are shown. (PDF 301 kb)

Additional file 6: **Figure S4.** Bacterial and fungal (A) α-diversity (local OTU richness); (B) γ-diversity (regional OTU richness); (C) β-diversity (unweighted Unifrac distance); (D) β-diversity (weighted Unifrac distance). Statistical analyses were performed independently for diversity indices at two seasons using Duncan’s multiple comparison test. The results were shown with colored letters a to d. Differences between samples of the same treatment at two seasons were performed using Tukey’s HSD test and indicated by symbols * (**: *P* < 0.001). (PDF 242 kb)

Additional file 7: **Figure S5.** Soil bacterial activity represented by the ratio of enzymatic activity to bacterial abundance under a temperature gradient. *n* = 1224 for each segmented graph (2 microcosm replicates for each treatment × 3 temperature levels × 17 soil samples per plot × 3 plot replicates × 4 grazing intensities = 1224 microcosms). Only the significantly correlated activity and bacterial abundance were calculated. (PDF 8671 kb)

Additional file 8: **Figure S6.** Soil bacterial activity represented by the ratio of enzymatic activity to bacterial abundance under a water content gradient. *n* = 1224 for each segmented graph (2 microcosm replicates for each treatment × 3 water content levels × 17 soil samples per plot × 3 plot replicates × 4 grazing intensities = 1224 microcosms). Only the significantly correlated activity and bacterial abundance were calculated. (PDF 8614 kb)

Additional file 9: **Figure S7.** Soil fungal activity represented by the ratio of enzymatic activity to fungal abundance under a temperature gradient. *n* = 1224 for each segmented graph (2 microcosm replicates for each treatment × 3 temperature levels × 17 soil samples per plot × 3 plot replicates × 4 grazing intensities = 1224 microcosms). Only the significantly correlated activity and bacterial abundance were calculated. (PDF 7855 kb)

Additional file 10: **Figure S8.** Soil fungal activity represented by the ratio of enzymatic activity to fungal abundance under a water content gradient. *n* = 1224 for each segmented graph (2 microcosm replicates for each treatment × 3 water content levels × 17 soil samples per plot × 3 plot replicates × 4 grazing intensities = 1224 microcosms). Only the significantly correlated activity and bacterial abundance were calculated. (PDF 8195 kb)
