# Supplementary figures and images for: Grazing-induced microbiome alterations drive soil organic carbon turnover and productivity in meadow steppe
Source: Microbiome. 2018 Sep 20;6:170. doi: 10.1186/s40168-018-0544-y (PMC6149009; doi:10.1186/s40168-018-0544-y)

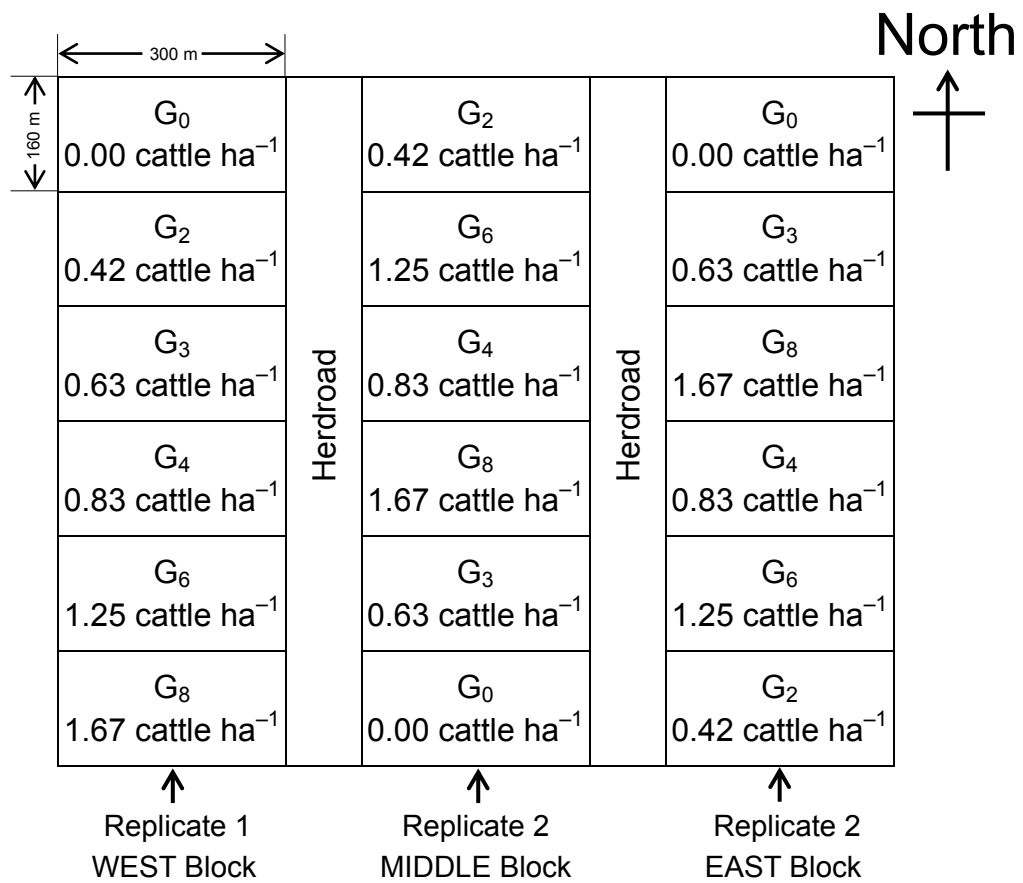

Supplement: Supplementary file 1 — Figure S1. Sketch map of the experimental site. (PDF 133 kb) [file 40168_2018_544_MOESM1_ESM.pdf]

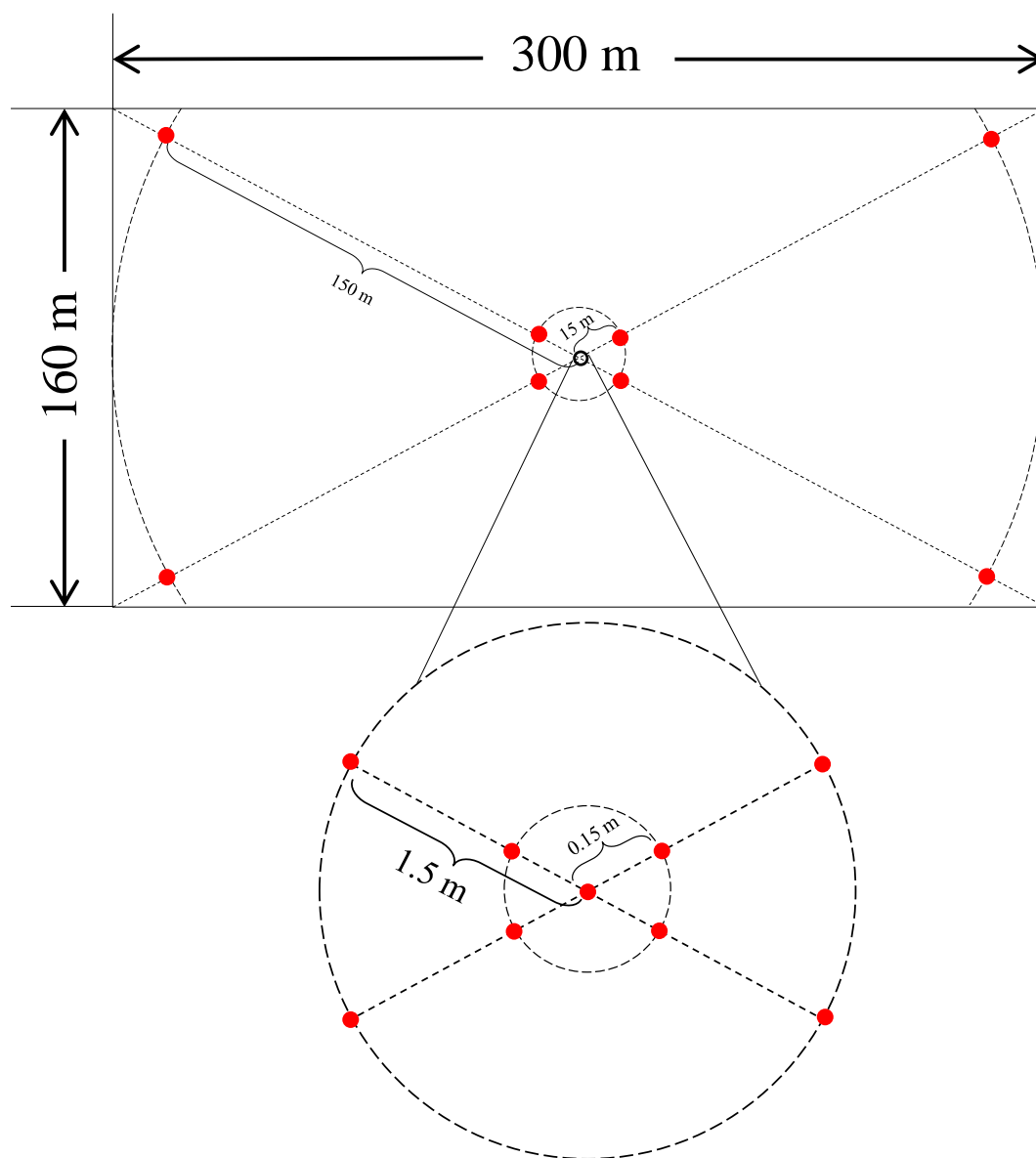

Supplement: Supplementary file 2 — Figure S2. Sampling scheme. A total of 17 soil cores (red points in the diagram) were taken from each plot. (PDF 18 kb) [file 40168_2018_544_MOESM2_ESM.pdf]

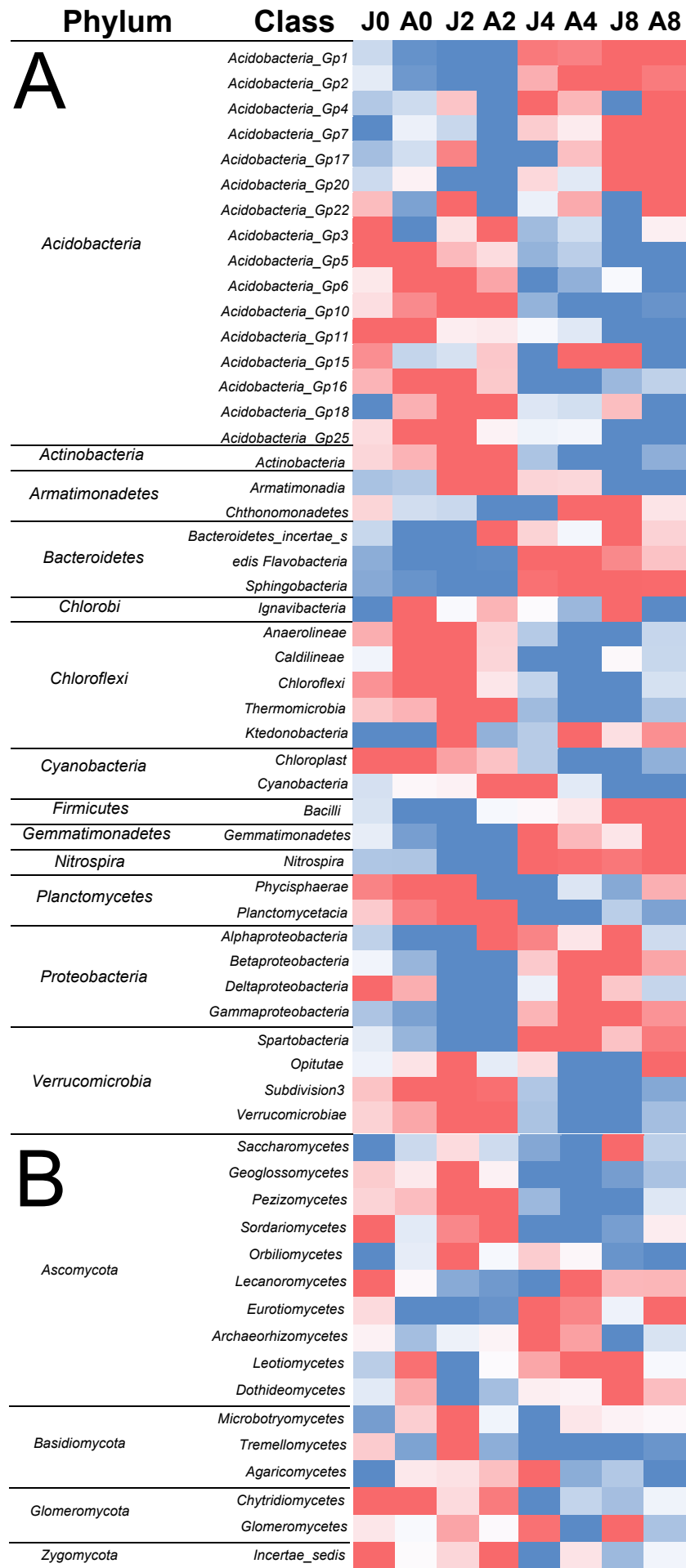

Supplement: Supplementary file 5 — Figure S3. Heatmap for (A) bacterial and (B) fungal communities. Color scale from greatest (red) to lowest (green) relative abundances within rows. Only the classifiable microbial classes are shown. (PDF 301 kb) [file 40168_2018_544_MOESM5_ESM.pdf]

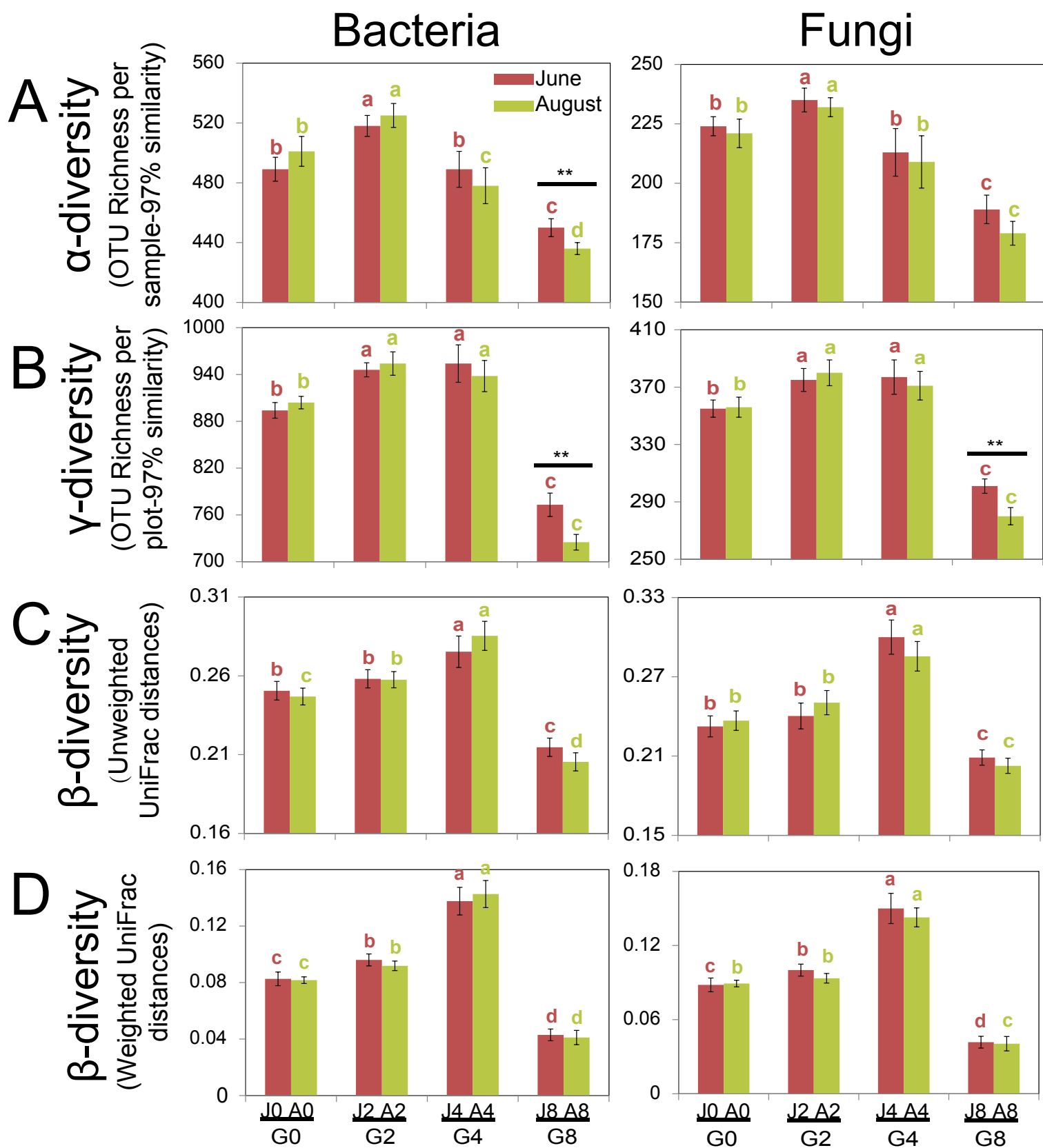

Supplement: Supplementary file 6 — Figure S4. Bacterial and fungal (A) α-diversity (local OTU richness); (B) γ-diversity (regional OTU richness); (C) β-diversity (unweighted Unifrac distance); (D) β-diversity (weighted Unifrac distance). Statistical analyses were performed independently for diversity indices at two seasons using Duncan’s multiple comparison test. The results were shown with colored letters a to d. Differences between samples of the same treatment at two seasons were performed using Tukey’s HSD test and indicated by symbols * (**: P < 0.001). (PDF 242 kb) [file 40168_2018_544_MOESM6_ESM.pdf]

50% Field capacity    75%Field capacity    100% Field capacity

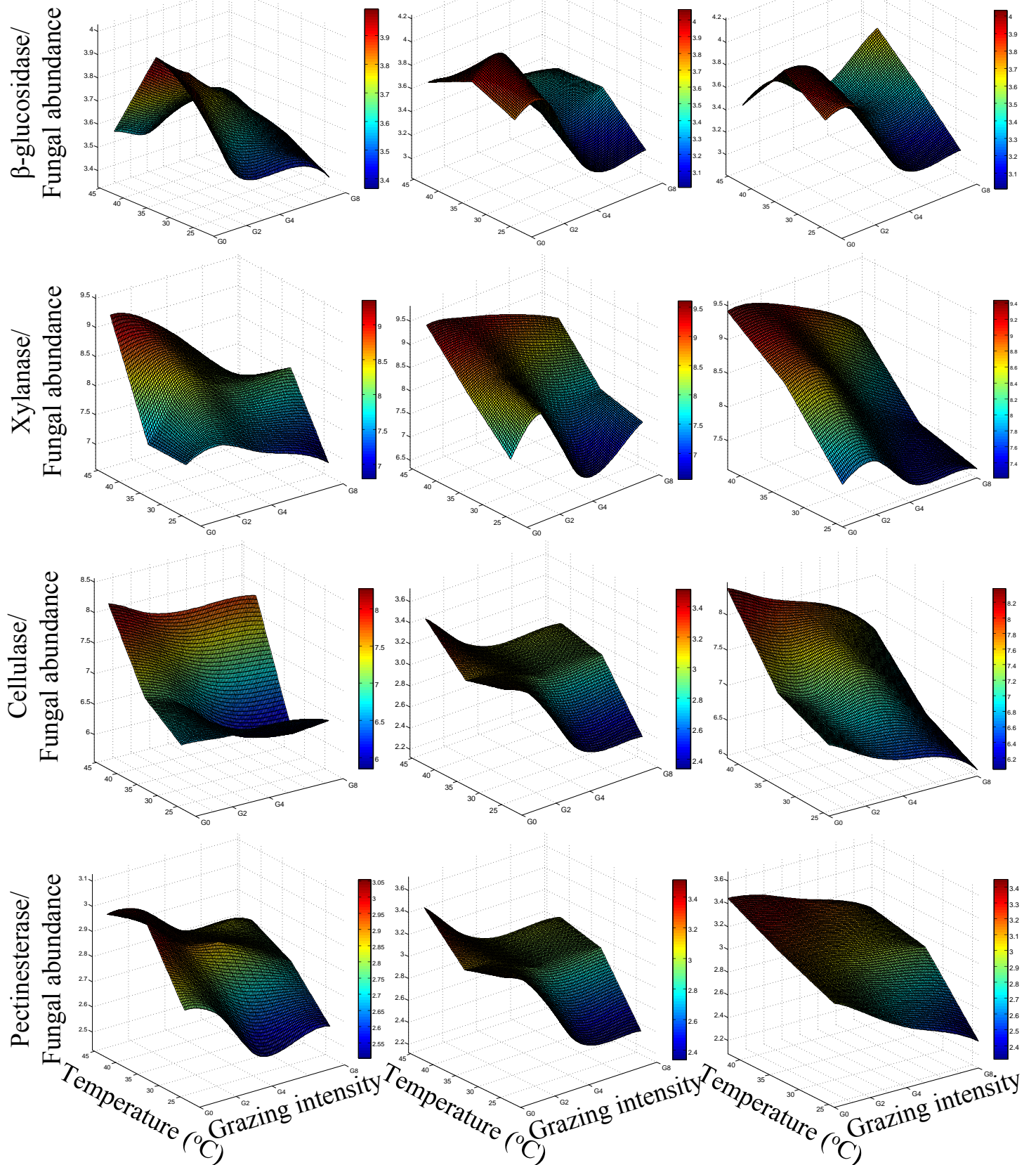

Supplement: Supplementary file 9 — Figure S7. Soil fungal activity represented by the ratio of enzymatic activity to fungal abundance under a temperature gradient. n = 1224 for each segmented graph (2 microcosm replicates for each treatment × 3 temperature levels × 17 soil samples per plot × 3 plot replicates × 4 grazing intensities = 1224 microcosms). Only the significantly correlated activity and bacterial abundance were calculated. (PDF 7855 kb) [file 40168_2018_544_MOESM9_ESM.pdf]

24 °C

33 °C

42 °C

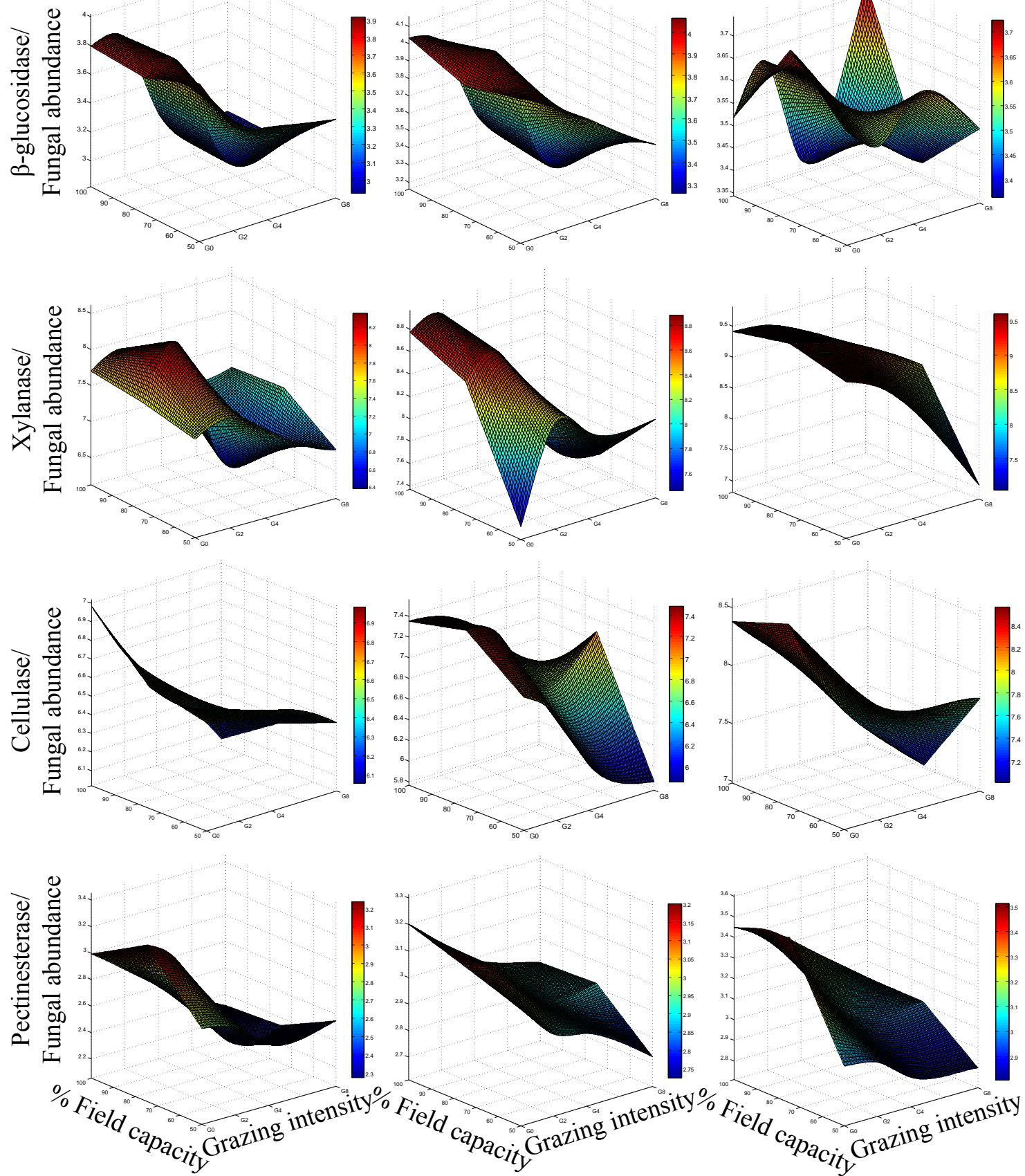

Supplement: Supplementary file 10 — Figure S8. Soil fungal activity represented by the ratio of enzymatic activity to fungal abundance under a water content gradient. n = 1224 for each segmented graph (2 microcosm replicates for each treatment × 3 water content levels × 17 soil samples per plot × 3 plot replicates × 4 grazing intensities = 1224 microcosms). Only the significantly correlated activity and bacterial abundance were calculated. (PDF 8195 kb) [file 40168_2018_544_MOESM10_ESM.pdf]
