# Supplementary material for: Grazing-induced microbiome alterations drive soil organic carbon turnover and productivity in meadow steppe
Source: Microbiome. 2018 Sep 20;6:170. doi: 10.1186/s40168-018-0544-y (PMC6149009; doi:10.1186/s40168-018-0544-y)
Supplement: Supplementary file 3 — Table S1. Soil water content (%) at sampling times (June and August, 2015). (DOCX 16 kb) [file 40168_2018_544_MOESM3_ESM.docx]

| Water capacity (%) | June | August |
| --- | --- | --- |
| G0 | 20.6±3.5 **a** | 22.6±3.3 **a** |
| G2 | 17.1±3.1 **a** | 19.5±4.2 **a** |
| G4 | 12.4±2.3 **b** | 13.4±1.3 **b** |
| G8 | 11.2±2.8 **b** | 12.1±2.2 **b** |

**Table S1** Soil water content (%) at sampling times (June and August, 2015)

Values represent mean ± standard deviation;

Different letters in column (shown in Bold) indicate significant differences (P < 0.05) among sites according to Duncan’s multiple comparison.
