# Supplementary material for: Grazing-induced microbiome alterations drive soil organic carbon turnover and productivity in meadow steppe
Source: Microbiome. 2018 Sep 20;6:170. doi: 10.1186/s40168-018-0544-y (PMC6149009; doi:10.1186/s40168-018-0544-y)
Supplement: Supplementary file 4 — Table S2. Detailed soil incubation conditions for moisture and/or temperature perturbance testing. (DOCX 16 kb) [file 40168_2018_544_MOESM4_ESM.docx]

**Table S2** Detailed soil incubation conditions for moisture and/or temperature perturbance testing

|  | | Temperature | | |
| --- | --- | --- | --- | --- |
|  |  | 24 ^o^C | 33 ^o^C | 42 ^o^C |
| Water content | 100% field capacity | 2 microcosms | 2 microcosms | 2 microcosms |
|  | 75% field capacity | 2 microcosms | 2 microcosms | 2 microcosms |
|  | 50% field capacity | 2 microcosms | 2 microcosms | 2 microcosms |
| Total |  | 18 microcosms | | |
