# Supplementary material for: Grazing-induced microbiome alterations drive soil organic carbon turnover and productivity in meadow steppe
Source: Microbiome. 2018 Sep 20;6:170. doi: 10.1186/s40168-018-0544-y (PMC6149009; doi:10.1186/s40168-018-0544-y)
Supplement: Supplementary file 7 — Figure S5. Soil bacterial activity represented by the ratio of enzymatic activity to bacterial abundance under a temperature gradient. n = 1224 for each segmented graph (2 microcosm replicates for each treatment × 3 temperature levels × 17 soil samples per plot × 3 plot replicates × 4 grazing intensities = 1224 microcosms). Only the significantly correlated activity and bacterial abundance were calculated. (PDF 8671 kb) [file 40168_2018_544_MOESM7_ESM.pdf]

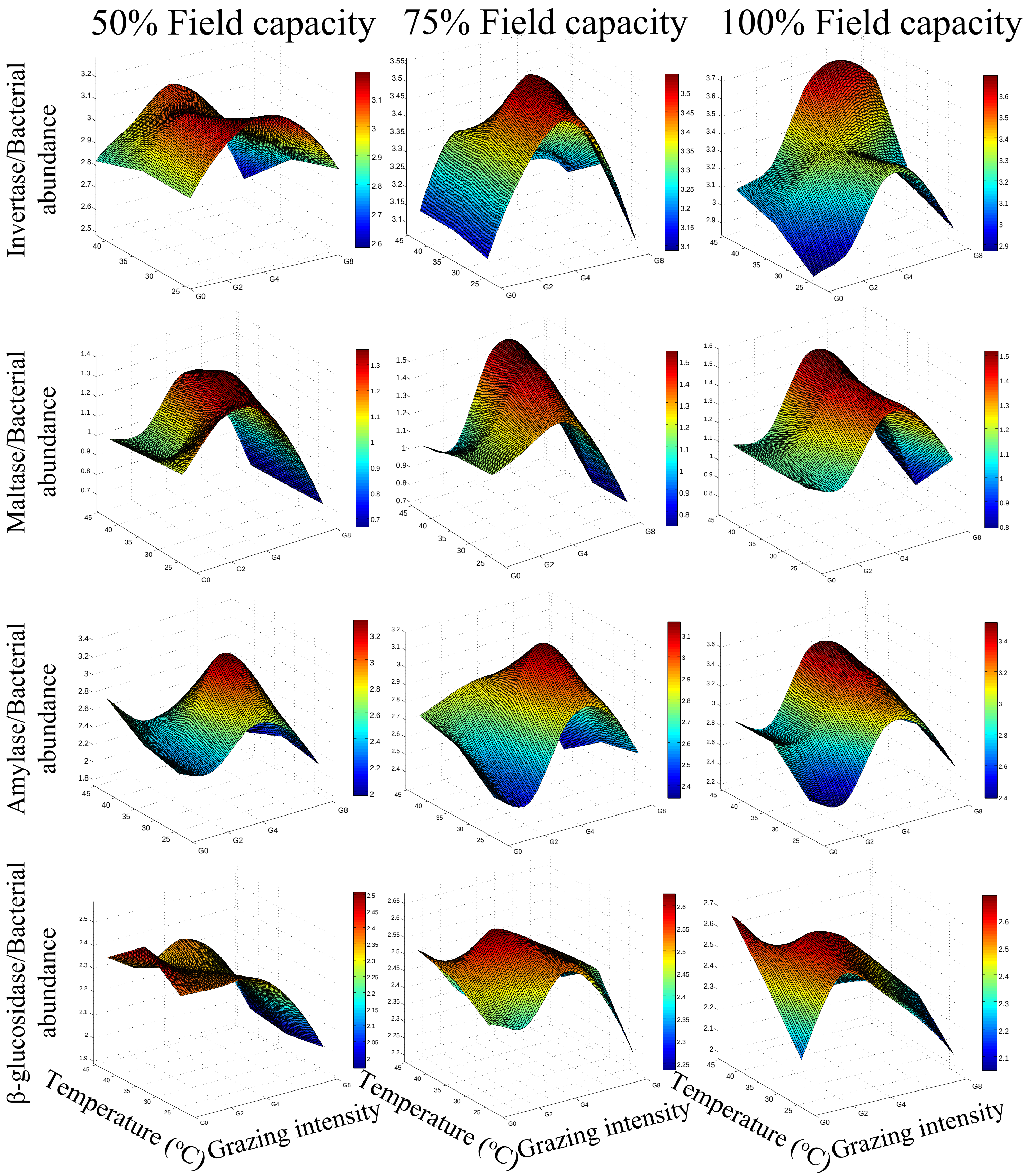
50% Field capacity
75% Field capacity
100% Field capacity
Invertase/Bacterial abundance
Maltase/Bacterial abundance
Amylase/Bacterial abundance
β-glucosidase/Bacterial abundance
Temperature (°C)
Grazing intensity
G0
G2
G4
G8
