# Supplementary material for: Grazing-induced microbiome alterations drive soil organic carbon turnover and productivity in meadow steppe
Source: Microbiome. 2018 Sep 20;6:170. doi: 10.1186/s40168-018-0544-y (PMC6149009; doi:10.1186/s40168-018-0544-y)
Supplement: Supplementary file 8 — Figure S6. Soil bacterial activity represented by the ratio of enzymatic activity to bacterial abundance under a water content gradient. n = 1224 for each segmented graph (2 microcosm replicates for each treatment × 3 water content levels × 17 soil samples per plot × 3 plot replicates × 4 grazing intensities = 1224 microcosms). Only the significantly correlated activity and bacterial abundance were calculated. (PDF 8614 kb) [file 40168_2018_544_MOESM8_ESM.pdf]

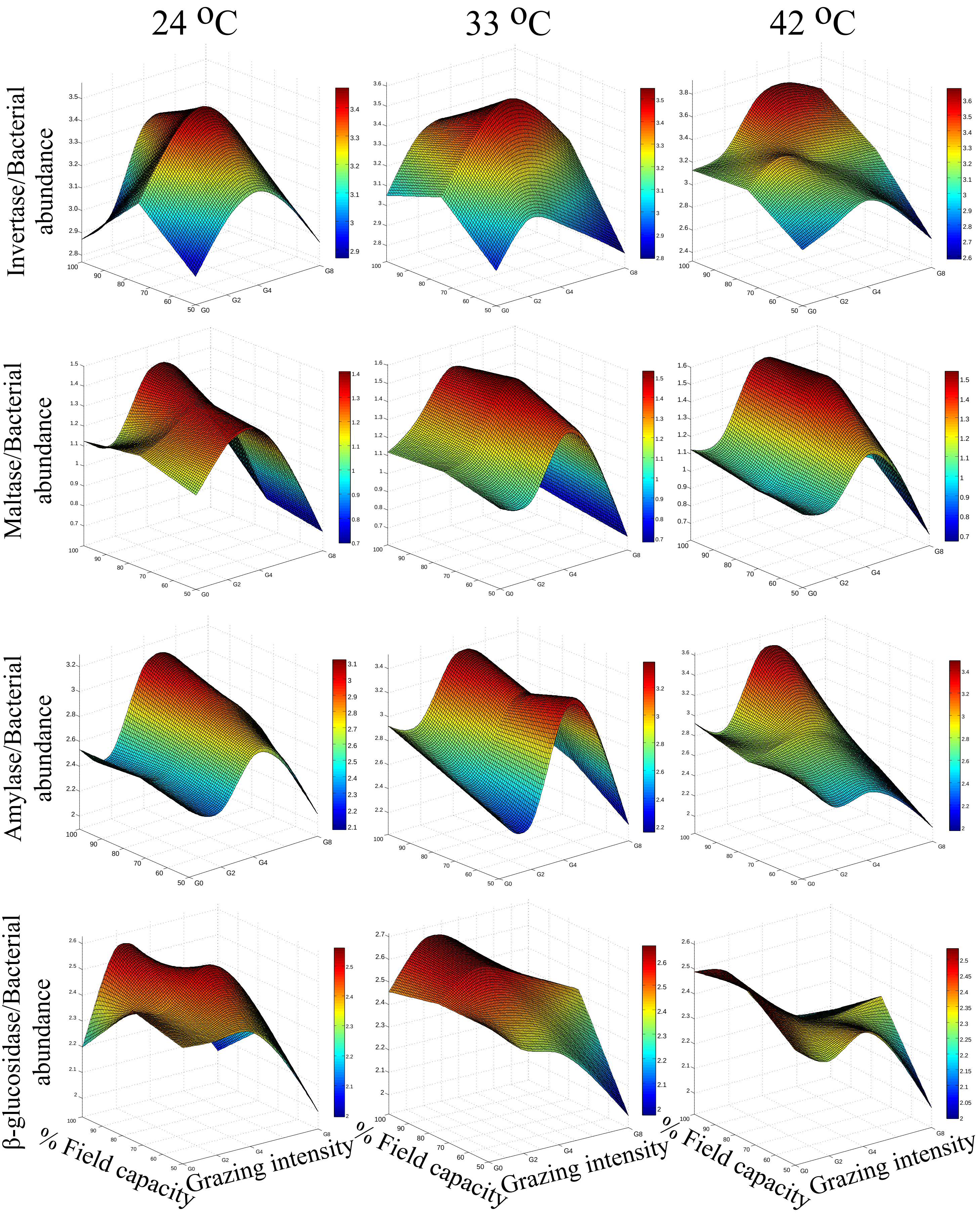

24 °C
33 °C
42 °C
Invertase/Bacterial abundance
Maltase/Bacterial abundance
Amylase/Bacterial abundance
β-glucosidase/Bacterial abundance
% Field capacity
Grazing intensity
G0
G2
G4
G8
100
90
80
70
60
50
